# Differences in health literacy domains among migrants and their descendants in Germany

**DOI:** 10.3389/fpubh.2022.988782

**Published:** 2022-09-23

**Authors:** Eva-Maria Berens, Julia Klinger, Sarah Carol, Doris Schaeffer

**Affiliations:** ^1^Interdisc. Cen. for Health Literacy Research, Bielefeld University, Bielefeld, Germany; ^2^Institute for Sociology and Social Psychology, University of Cologne, Köln, Germany; ^3^School of Sociology, University College Dublin, Dublin, Ireland

**Keywords:** health literacy, determinants and correlates, immigration and migration, Turkey, former Soviet Union (FSU), Russian-speaking, self-efficacy, interethnic contact

## Abstract

**Background:**

Health literacy (HL) is considered to be an important precondition for health. HL research often identifies migrants as vulnerable for low HL. However, in-depth data on HL among migrants especially in its domains of health care, disease prevention and health promotion and its determinants are still scarce.

**Objective:**

The aim of this study was therefore to analyse the current status of HL among migrants and their descendants from Turkey and from the former Soviet Union (FSU) in Germany and factors associated with it. This has not been studied using large-scale data and bilingual interviews. We differentiate between dimensions of HL, namely the domains of health care, disease prevention and health promotion which goes beyond many previous studies. In addition, we explore new mechanisms by testing the explanatory power of self-efficacy and interethnic contacts for migrants' HL.

**Methods:**

The study includes 825 first- and second-generation adult migrants from two of the largest immigration groups in Germany, from Turkey and FSU, who were interviewed face-to-face in German, Turkish or Russian in late summer 2020. HL was measured using the HLS_19_-Q47 instrument. Age, gender, educational level, social status and financial deprivation, chronic illness, health-related literacy skills, self-efficacy, interethnic contacts, migration generation, duration of stay and region of origin were considered as possible determinants. Ordinary least square regressions were estimated.

**Results:**

The average general HL score was 65.5. HL in health promotion and disease prevention was lower than in health care. Low financial deprivation, health-related literacy skills, and self-efficacy were positively correlated with each HL domain. Educational level, social status, age, gender, duration of stay and interethnic contacts were positively correlated with HL in some domains. Region of origin was only correlated with the domain of disease prevention until interethnic contact was accounted for.

**Conclusion:**

Our study contributes to the existing knowledge by analyzing different domains of HL and testing its correlations with self-efficacy and interethnic contact among migrants. We reveal that migrants cannot generally be considered as vulnerable for low HL, as oftentimes outlined. There is a need for interventions e.g. to enhance the understanding of health information among subgroups with lower HL.

## Introduction

Health literacy, understood as the ability to find, understand, assess and apply health-related information to make appropriate health decisions and take action in everyday life concerning health care, disease prevention and health promotion (1), has become an increasingly important topic in public health research and policy (2). It has widely been shown to be associated with poor health behavior, poor health outcomes, lower use of preventive measures, and increased use of several health services [ex. (3–6)], leading to additional annual health care costs (7). The concept of health literacy is relational; thus a person's health literacy depends on both the individual skills and abilities as well as situational demands (1). In other words, the need and the quality of individual skills and abilities depend on organizational structures and access to reliable health information presented in an understandable, assessable and applicable manner.

Recent surveys have shown that large proportions of adult populations, including adults from Germany, have limited health literacy (5, 6, 8, 9). Studies have also highlighted that health literacy is unequally distributed. Several groups, such as, for instance, persons with lower socioeconomic status, have particularly low health literacy [ex. (6)] meaning that it is more difficult for them to deal with health-related information. It has also been indicated that migrant populations have particularly low health literacy. The term “migrant” in this paper generally refers to persons who migrated themselves across international borders and their descendants born in the country of residence who are labeled as second generation without any limitations regarding reasons for moving, duration of staying and residence status. Research in the United States, Canada and Australia mostly shows large proportions of low health literacy among ethnic minorities or migrants, including Asians, Latin Americans, former Soviet Union migrants and other ethnic groups (10–14). In Europe, this includes, for instance, migrants with refugee status and Arabic-speaking migrants in Sweden (15, 16), women from Somalia and different migration groups in Norway (17, 18), Russian-speaking migrants in Israel (19), Turkish, Portuguese and former Yugoslavian migrants in Switzerland and Austria (20, 21). To the best of our knowledge, research on migrants in Germany is scarce. Existing datasets include only a small number of migrants, which does not allow for detailed analyses [ex. (8, 22, 23)]. Our aim is to fill this research gap.

The factors associated with low health literacy among migrant and non-migrant populations are manifold, especially when a broad socio-ecological and relational concept of health literacy is considered. Literacy skills in general, sometimes framed as functional health literacy and included as a basic prerequisite in many health literacy definitions [ex. (1, 24)], are strongly associated with health literacy [ex. (8, 22, 25, 26)]. Furthermore, lower socioeconomic or social status and lower levels of formal education are associated with low health literacy (4–6, 8, 9, 12, 13, 19, 22, 27–32). Several studies also indicate that health literacy mostly declines with increasing age, is sometimes associated with gender without a consistent pattern, and in some cases lower among persons with chronic diseases (4, 6, 8, 29, 33). More recently, psychological aspects gained attention in health literacy research. For example, self-efficacy, defined as ‘beliefs in one's capabilities to organize and execute the courses of action required to produce given attainments' (34), was found to be linked to health literacy in the general population (35–37). This seems plausible as dealing with health information might be complicated and self-efficacy in other words is the subjective certainty of being able to cope with new and difficult situations. In addition, psychological characteristics can affect competences more directly than sociodemographic and -economic factors. Thus, this might be a relevant aspect for health literacy interventions. In addition, scholars demand more focus on the system and social-ecological context in which health information is provided instead of individual skills (38). In this regard, individuals' social ties are expected to be related to health literacy (30, 39). Among migrants, especially social ties with persons that are familiar with the national health system and thus social integration in form of interethnic contacts might be relevant for their health literacy. Interethnic friendships are a common indicator of social integration (40). Qualitative studies support the relevance of these aspects (41–43). To the best of our knowledge, there are no quantitative studies on the association of self-efficacy and interethnic contacts with health literacy among migrants so far. Our study attempts to fill this gap. We consider it to be important to investigate health literacy more holistically by identifying factors that lie within the migrants' environment and network, as well as individual characteristics such as self-efficacy, which imply that migrants are also self-determined actors.

Additionally, migrant-specific aspects, such as low proficiency in the receiving country's language and a shorter duration of stay have been identified as factors associated with low health literacy (12, 15, 16, 18, 21, 32, 44–46). Research considering second generation migrants is scarce (21). Several studies also indicate differences in health literacy by migrant's region or country of origin. For example, in Switzerland, Austria and Norway, migrants from Turkey had lower health literacy than other immigration groups (18, 20, 21). In a sample of older migrants in a federal state in Germany (North Rhine Westphalia), migrants form Turkey and Poland had lower health literacy in the domain of health care than migrants from Italy and Greece (32).

Another gap in the literature concerns a differentiation between domains of health literacy to enable conclusions for specific contexts of health information. Although the general health literacy measurement (HLS-EU-Q47) used in many European studies (47) would allow for domain-specific analyses, there are only a few studies reporting health literacy of the general population in its domains of health care, disease prevention and health promotion. They show differences in health literacy between the domains. In most studies, health literacy in the domain of health promotion was lowest (5, 20, 48, 49). Comparing health care and disease prevention domains did not reveal a consistent pattern (5, 20, 33, 49, 50). Detailed information on health literacy in different domains among migrants is rare and so far only considered in national reports. In Switzerland, Portuguese and Turkish migrants showed the lowest health literacy in the domain of disease prevention and health promotion (20). In Norway, differences across domains were very small among immigration groups (18). In a small diverse migrant sample in Portugal, health care and disease prevention literacy were similarly pronounced but health promotion literacy was substantially lower (51). Studies comparing determinants of health literacy across domains, especially among migrants, are scarce (18, 33, 52). We address this research gap in our study.

It is crucial to know more about health literacy in different population groups and especially about health literacy in its domains in order to be able to design interventions, which can improve the provision of information to migrants. The aim of this article is therefore to analyse health literacy focusing specifically on the domains of health care, disease prevention and health promotion among migrants in Germany. Furthermore, we add migrant-specific, demographic, socioeconomic and psychological variables (self-efficacy) as well as social ties (interethnic contacts) to the equation.

In our analyses, we focus on two of the largest immigration groups in Germany, Turkish migrants and migrants from the former Soviet Union (FSU) and their descendants. In 2021, around 3 million people of Turkish descent and around 3.5 million people from the former Soviet Union live in Germany (53). Most of the Turkish migrants came as guest workers (temporary labor migrants) following an agreement with Turkey in 1961, followed by family reunions. Many of FSU migrants arrived in the late 1980s from Russia but are ethnic Germans (*Aussiedler*). The socioeconomic status of migrants is, on average, lower than that of natives. However, FSU migrants have a higher socioeconomic status than Turkish migrants (54, 55), partly due to the recognition of their degrees (56) or a linguistic advantage among ethnic Germans.

The following questions are addressed:

- What is the current status of health literacy among migrants (i.e., from Turkey and from FSU) in Germany in general and in three different domains of health care, disease prevention and health promotion?
- Which factors are associated with health literacy in general and the three domains?
  ° Are there differences in health literacy by migrant-specific aspects, i.e., country of origin and generation/duration of stay?
  ° Which role do demographic, socioeconomic, health-related aspects and health-related literacy skills play?
  ° Which role do psychological aspects (self-efficacy) and social ties (interethnic contacts) play?

## Materials and methods

### Data

Data from the survey “Health literacy survey of people with migration background in Germany” (HLS-MIG) were used for this article. It is related to the second health literacy survey in Germany [HLS-GER 2 (49)]. The data were collected and stored anonymously in compliance with ESOMAR (European Society for Opinion and Marketing Research) standards and European GDPR (General Data Protection Regulation).

The respondents of the quantitative cross-sectional survey were persons at least 18 years old and who themselves or at least one of their parents have / had a citizenship from Turkey or a country belonging to the former Soviet Union. The survey was conducted in August/September 2020 using paper-assisted oral-personal interviews (PAPI). The interview was offered in German, Turkish and Russian (57).

Multi-stage random and quota sampling was used. At first, sampling points were selected within most of the federal states of Germany with a probability proportional to the targeted population size in the respective federal states. For each selected sampling point one bilingual interviewer was recruited who in turn recruited respondents by means of nationwide quotas regarding age, gender and personal or parental (one- or two-sided) migration experience, i.e., first- or second-generation migrants, for each immigration group based on official statistics (53). After exclusion of 21 interviews with less than 80% of the core questions answered as well as interviews with unusually monotonous response behavior or unusual similarity of the interviews of a specific interviewer, a total of 1,037 respondents were included in the original study.

### Measures

Health literacy was measured with the instrument HLS_19_-Q47(-DE), which is based on the HLS-EU-Q47 that was revised and refined for the Health Literacy Population Survey 2019–2021 (6). Following Sorensen's et al. (1) health literacy definition, it contains 47 items on specific health information tasks in the domains of health care (16 items), disease prevention (15 items), and health promotion (16 items) and the information management steps accessing, understanding, appraising, and applying. The respondents were asked to assess perceived difficulties with the options “very easy,” “easy,” “difficult,” and “very difficult”. In this article, the items were combined into four health literacy scores, one for each domain and one for general health literacy. In accordance with the HLS_19_ procedure, each score is calculated as the percentage of items that were answered with “very easy” or “easy” if 80 % of the corresponding items were answered (6). Therefore, scores range from 0 to 100 and higher values signify a higher level of health literacy.

Two migration-specific factors were included. Region of origin was coded into 0 “FSU” and 1 “Turkey.” Duration of stay was combined with migration generation to include respondents that were born in Germany in the same models: “second generation,” “up to 5 years,” “6 to 25 years,” “26 years and more.”

Demographic and socioeconomic factors, age, gender, educational level, social status, and financial deprivation were used. We took age measured in years and gender (1 “female” and 0 “male”) into account. Educational level was categorized according to the International Standard Classification of Education [ISCED-11 (58)], ranging from 0 “no formal education” to 8 “doctorate.” Due to low case numbers for the lowest category (*n* = 7), it was combined with ISCED level 1 (primary education). To facilitate the allocation, respondents were asked about their school and vocational degrees, including those that are or were possible to achieve in Turkey and FSU countries. Social status was operationalized with a question on the self-perceived position in society, using the image of a ladder, ranging from 1 to 10. Respondents were asked “On the following scale,” step ‘1' corresponds to “the lowest level in the society”; step ‘10' corresponds to “the highest level in the society.” Could you tell me on which step you would place yourself?” (59). Financial deprivation was based on the perceived difficulty in paying for medications with answers “very easy” = 1 “none,” “easy” = 2 “low,” “difficult” and “very difficult” = 3 “medium–high,” the latter two being combined due to low case numbers for the last category (*n* = 28). As a health-related aspect, chronic disease was included. A chronic disease was ascribed to respondents with one or several chronic diseases (for at least 6 months) (0 “no,” 1 “yes”). German health-related literacy skills were included based on the objective Newest Vital Sign test. The test measures the ability to read and apply information from an ice cream nutrition label and comprises six questions. The sum score of correct answers varies from 0 to 6, with a higher value for higher health-related literacy skills (60). The accompanying food label was provided in German only to test German specific literacy skills.

Self-efficacy was measured with a validated short version instrument (ASKU) with 3 items: (i) I can rely on my own abilities in difficult situations. (ii) I am able to solve most problems on my own. (iii) I can usually solve even challenging and complex tasks well. Respondents could answer on a five-point Likert scale ranging from 1 “does not apply at all” to 5 “applies completely”. The items were combined in a mean score ranging from 1 to 5 with a higher value for higher self-efficacy (61). Interethnic contact was operationalized with a question on the share of friends or acquaintances of German origin with 5 categories (1 “none/almost none,” 2 “less than half,” 3 “half,” 4 “more than half” and 5 “all or almost all”).

### Statistical analyses

Ordinary least square (OLS) regressions were estimated by health literacy domain. Overall, five models were estimated. The null model (M1) only contains region of origin. In Model 2, we add generation, in Model 3 demographic and socioeconomic variables, in Model 4 self-efficacy and in the last model interethnic contact. In addition, ANOVAs were used to indicate statistically significant differences between subgroups, based on the 95% confidence intervals. We calculated clustered standard errors on the level of interviewers (*n* = 113) to account for interviewer effects (62). We applied listwise deletion based on missing values of at least one of the described variables with 825 respondents remaining in the sample. There were no statistically significant differences in health literacy scores in the full and restricted sample.

## Results

### Characteristics of the study sample

As shown in Table 1, the mean age of the sample was almost 44 years and more than half was female or had at least one chronic disease. Around one third had high education and around two-thirds had high health-related literacy skills. The social status was mostly perceived to be in the medium categories and one half had low financial deprivation. Self-efficacy in the sample was rather high. One quarter of the sample was born in Germany and for first generation migrants the duration of stay was on average 22 years. The majority of the sample has interethnic contacts with persons of German origin. There were substantial differences between FSU and Turkish migrants regarding educational level, age, financial deprivation, the proportion of first generation and duration of stay (see Supplementary Table 1). Most people of the FSU sample were born in Russia, Kazakhstan or Ukraine.

**Table 1:** Sample characteristics.

	***n***	**%**		***n***	**%**
**Region of origin**			**Financial deprivation**		
Former Soviet Union	413	50.1	None	195	23.6
Turkey	412	49.9	Low	442	53.6
**Duration of stay**			Medium–high	188	22.8
Mean (SD), min–max	22.4 (13.8)	0–58	**Chronic disease(s)**
5 years and below	99	12.0	Yes	447	54.2
6–25 years	282	34.2	No	378	45.8
26 years and longer	219	26.5	**Health-related literacy skills**
Born in Germany	225	27.3	Mean (SD), min–max	3.9 (1.8)	1–6
**Gender**			Low (NVS 0–1)	107	13.0
Male	380	46.1	Medium (2–3)	195	23.6
Female	445	53.9	High (4–6)	523	63.4
**Age**			**Self-efficacy**
Mean (SD), min–max	43.5 (16.2)	18–91	Mean (SD), min–max	3.9 (0.8)	1–5
18–29 years	202	24.5	Low (below mean)	331	40.1
30–45 years	278	33.7	High (above mean)	494	59.9
46–64 years	242	29.3	**Interethnic contacts**
65 years and older	103	12.5	Mean (SD), min–max	2.8 (1.3)	1–5
**Educational level**			None or almost none	150	18.2
Low (ISCED 0–2)	185	22.4	Less than half	232	28.1
Medium (3–4)	333	40.4	Around half	186	22.5
High (5–8)	307	37.2	More than half	175	21.2
**Social Status**			All or almost all	82	9.9
Mean (SD), min–max	5.7 (1.7)	1–10			
Low (1–4)	161	19.5			
Medium (5–7)	545	66.1			
High (8–10)	119	14.4			

### Descriptive statistics on health literacy domains

**Figure 1:**
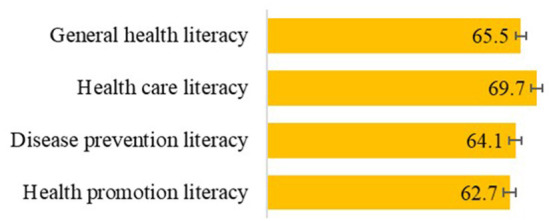
Health literacy score means (means with range 0–100 and 95 % confidence intervals).

As shown in Figure 1, the mean general health literacy score among migrants in the sample was 65.5 out of 100 (95% CI: 64.1, 66.9). Significant differences were found between the three domains of health literacy. Health literacy in the domains of disease prevention [64.1 (62.5, 65.8)] and health promotion [62.7 (61.1, 64.2)] were significantly lower than in the domain of health care [69.7 (68.1, 71.2)]. This means that more health information tasks regarding disease prevention and health promotion were perceived as (very) difficult compared to information regarding health care.

**Figure 2:**
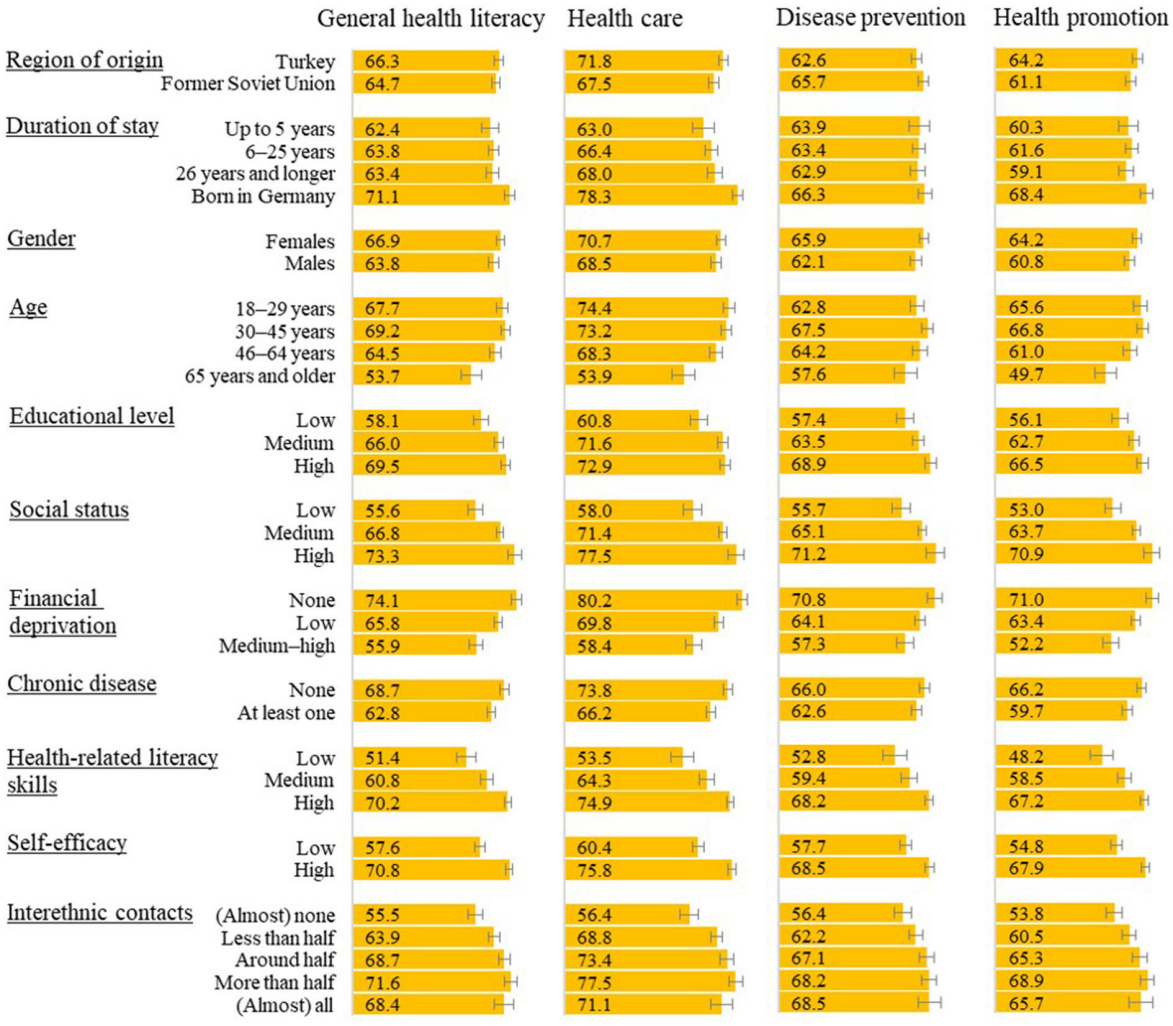
Health literacy score means in subgroups (means with range 0–100 and 95% confidence intervals).

Bivariate analyses showed that health information was more difficult to process for specific subgroups (Figure 2). Overall, there were differences in health literacy scores regarding migration-specific factors, demographic, socioeconomic, health and literacy related, self-efficacy and interethnic contacts. FSU migrants in our sample had a slightly lower score in the domains of health care (*p* < 0.05) and health promotion than migrants from Turkey. Migrants from Turkey in turn had a slightly lower score in disease prevention. Persons who were born abroad had lower scores, especially in the domains of health care (*p* < 0.05) and health promotion. Health literacy scores across all domains were also lower among older respondents. Respondents with at least one chronic disease had a lower health literacy score. This also applied to respondents with a lower educational level or health-related literacy skills. A lower social status and financial deprivation were reflected in a lower health literacy score as well. Finally, those with lower self-efficacy and those with fewer interethnic contacts had a lower health literacy score.

### Determinants of health literacy domains

Results of the ordinary least square regressions (Table 2) showed that r*egion of origin* was only significantly associated with health literacy in the domain of disease prevention, with lower values for Turkish migrants when controlling for demographic and socioeconomic factors, literacy, chronic illness and self-efficacy (model 4). After adding interethnic contacts (model 5), the association with region of origin decreases and is no longer significant. Lower health literacy in the domain of health care among FSU migrants was only present in the null model (model 1) and the contrast disappeared when taking duration of stay into account. No correlation with region of origin were found for general health literacy and in the domain of health promotion. All categories of the *duration of stay* (reference second-generation migrants) were significantly connected with health literacy in almost all domains (model 2), thus first-generation migrants had a lower health literacy compared to immigrant descendants. When controlled for demographic and socioeconomic factors, literacy and chronic illness (model 3), a shorter duration of stay (up to 5 years) remained significantly associated with a lower general health literacy and in the domain of health care.

**Table 2:** Factors associated with health literacy—stepwise ordinary least square (OLS) regression results.

	**General health literacy**					**Health care literacy**					**Disease prevention literacy**					**Health promotion literacy**				
	**M1**	**M2**	**M3**	**M4**	**M5**	**M1**	**M2**	**M3**	**M4**	**M5**	**M1**	**M2**	**M3**	**M4**	**M5**	**M1**	**M2**	**M3**	**M4**	**M5**
Constant	**64.74**	**72.50**	**60.12**	**36.92**	**32.35**	**67.47**	**79.01**	**67.847**	**40.24**	**35.87**	**65.68**	**70.81**	**53.92**	**34.50**	**30.68**	**61.12**	**67.59**	**58.40**	**36.15**	**30.26**
	*1.383*	*2.308*	*5.076*	*7.042*	*7.392*	*1.567*	*2.34*	*5.139*	*7.035*	*7.522*	*1.577*	*2.851*	*6.203*	*8.183*	*8.586*	*1.558*	*2.646*	*6.573*	*8.292*	*8.372*
Region of origin: Turkey	**1.546**	**−1.669**	**−1.482**	**−1.248**	**−0.260**	4.363^*^	**−0.834**	**−0.960**	**−0.681**	**0.264**	**−3.084**	−5.407^**^	−4.729^*^	−4.533^*^	**−3.678**	**3.059**	**0.979**	**1.014**	**1.239**	**2.329**
(ref. former Soviet Union)	*2.055*	*2.402*	*2.141*	*2.113*	*2.130*	*2.247*	*2.530*	*2.128*	*2.094*	*2.101*	*2.361*	*2.657*	*2.498*	*2.497*	*2.562*	*2.204*	*2.677*	*2.552*	*2.512*	*2.519*
Duration: up to 5 years^∧^		−9.79^***^	−5.355^**^	**−5.074**	**−3.264**		−15.90^***^	−10.97^***^	−10.64^***^	−8.905^**^		−5.740^*^	**−1.876**	**−1.641**	**−0.11**		−7.51^**^	**−3.034**	**−2.764**	**−1.353**
		*3.139*	*2.923*	*3.028*	*3.143*		*3.816*	*3.302*	*3.432*	*3.491*		*3.456*	*3.549*	*3.617*	*3.730*		*3.304*	*3.160*	*3.243*	*3.516*
6–25 years^∧^		−8.26^***^	**−1.671**	**−1.528**	**−0.631**		−12.4^***^	−3.675^*^	**−3.504**	**−2.647**		−6.044^**^	**−2.242**	**−2.121**	**−1.427**		−6.20^**^	**0.848**	**0.985**	**1.763**
		*2.267*	*2.170*	*2.103*	*2.049*		*2.412*	*2.171*	*2.127*	*2.133*		*2.753*	*2.738*	*2.739*	*2.715*		*2.627*	*2.625*	*2.499*	*2.441*
26 years and more^∧^		−8.13^***^	**1.356**	**−0.008**	**0.122**		−10.5^***^	**2.582**	**0.959**	**1.084**		**−4.554**	**0.249**	**−0.893**	**−0.821**		−9.07^***^	**1.194**	**−0.113**	**0.017**
		*2.647*	*2.363*	*2.309*	*2.291*		*2.706*	*2.420*	*2.373*	*2.355*		*3.185*	*3.162*	*3.101*	*3.094*		*2.691*	*2.605*	*2.570*	*2.574*
Females			**2.264**	2.982^**^	2.922^**^			**1.318**	**2.173**	**2.116**			2.971^*^	3.572^**^	3.530^**^			**2.53**	3.219^**^	2.999^*^
(ref. males)			*1.378*	*1.393*	*1.373*			*1.519*	*1.543*	*1.525*			*1.724*	*1.766*	*1.739*			*1.548*	*1.531*	*1.521*
Age			−0.171^**^	−0.119^*^	**−0.107**			−0.305^***^	−0.243^***^	−0.232^***^			**0.008**	**0.052**	**0.063**			−0.206^***^	−0.156^**^	**−0.131**
			*0.069*	*0.068*	*0.070*			*0.077*	*0.074*	*0.075*			*0.081*	*0.081*	*0.082*			*0.076*	*0.077*	*0.079*
Educational level			1.456^***^	0.963^**^	0.907^**^			1.694^***^	1.108^**^	1.054^**^			1.127^*^	**0.714**	**0.672**			1.536^***^	1.063^**^	0.948^**^
			*0.429*	*0.394*	*0.392*			*0.493*	*0.446*	*0.439*			*0.494*	*0.464*	*0.465*			*0.457*	*0.445*	*0.446*
Social status			1.630^***^	1.148^**^	1.086^**^			1.993^***^	1.419^***^	1.360^**^			1.418^*^	**1.014**	**0.962**			1.450^***^	0.988^*^	0.939^*^
			*0.486*	*0.494*	*0.496*			*0.525*	*0.533*	*0.544*			*0.644*	*0.697*	*0.696*			*0.539*	*0.507*	*0.500*
Financial deprivation			−4.921^***^	−3.897^***^	−3.649^***^			−4.791^***^	−3.572^***^	−3.335^***^			−4.760^***^	−3.903*^*^	−3.682*^*^			−5.211^***^	−4.229*^*^	−3.822*^*^
			*1.275*	*1.263*	*1.275*			*1.279*	*1.233*	*1.228*			*1.507*	*1.532*	*1.552*			*1.640*	*1.624*	*1.635*
Chronic disease(s)			**−0.337**	**−0.095**	**0.012**			**−0.179**	**0.108**	**0.211**			**−0.368**	**−0.166**	**−0.038**			**−0.477**	**−0.245**	**−0.212**
(ref. none)			*1.477*	*1.501*	*1.499*			*1.582*	*1.592*	*1.594*			*1.933*	*1.976*	*1.961*			*1.767*	*1.767*	*1.745*
Health–related literacy skills			2.059^***^	1.836^***^	1.768^***^			2.098^***^	1.834^***^	1.768^***^			2.205^***^	2.018^***^	1.942^***^			1.867^***^	1.653^***^	1.642^***^
			*0.498*	*0.473*	*0.469*			*0.543*	*0.500*	*0.501*			*0.601*	*0.586*	*0.584*			*0.571*	*0.559*	*0.551*
Self–efficacy				6.231^***^	6.115^***^				7.415^***^	7.303^***^				5.217^***^	5.077^***^				5.975^***^	5.835^***^
				*1.022*	*1.010*				*1.154*	*1.140*				*1.229*	*1.218*				*1.073*	*1.055*
Interethnic contacts					1.358*^*^					1.299^*^					**1.180**					1.572*^*^
					*0.559*					*0.669*					*0.748*					*0.628*
Corrected. R^2^	0.0002	0.025	0.215	0.266	0.271	0.008	0.054	0.256	0.315	0.318	0.003	0.008	0.105	0.129	0.131	0.003	0.020	0.163	0.199	0.205

N = 825; bold numbers: B-coefficients; italic numbers: standard errors;

*** p < 0.01,

** p < 0.05

* p < 0.1;

∧ reference (ref.): born in Germany; M: Model.

As shown in model 3, overall, a higher *educational level, higher social status* and *better health-related literacy skills* are linked to a higher health literacy, whereas *financial deprivation* and increasing *age* (except for disease prevention) are significantly associated with a lower health literacy in all domains. Having at least one c*hronic disease* is not associated with health literacy in any domain in this sample. Women tend to have a higher health literacy.

*Self-efficacy* (model 4) was significantly associated with health literacy in all domains. Respondents with a higher self-efficacy had a higher health literacy. It should be stressed that self-efficacy considerably decreases the correlations of the other independent variables with the dependent variables in the model. Furthermore, adding *interethnic contacts* (model 5) showed statistically significant and positive associations with health literacy in all domains, except for disease prevention.

The full model (model 5) explained 13.2 % (disease prevention) to 32.0 % (health care) of the total variance of health literacy. The models with only region of origin and duration of stay (model 1 and 2) explained around 1 to 6% of the total variance. Including demographic and socioeconomic aspects, health-related literacy skills and chronic disease (model 3) explained considerably more variance, and the model including self-efficacy (model 4) further increases explained variance. Adding interethnic contacts does not add much explanation of variance.

## Discussion

A large proportion of the population in various countries is experiencing substantial difficulties in dealing with health information [ex. (6, 9, 47)]. Thus, improving health literacy has become an important focus of health policies in many countries (2), including Germany (63). In order to design interventions that can improve health literacy, knowledge about health literacy in potentially vulnerable population groups such as migrants is crucial. The aim of this study was therefore to analyze the current status of health literacy among migrants and their descendants (i.e., from Turkey and from FSU) in Germany and factors associated with it. Our contribution to the current state-of-the-art is threefold: First, our study goes beyond existing studies on migrant populations by drawing on a newly collected, larger dataset and also include the non-German speaking population. To date, there were only some qualitative studies taking a deeper look into the matter (41–43, 64). Existing quantitative research on health literacy of migrants in Germany could only analyse rather small subsets of migrants from diverse backgrounds with good German language skills or subgroups such as poorly educated adolescents or elderly (8, 23, 32, 50). Second, going beyond many previous studies, we differentiate between dimensions of health literacy, namely the domains of health care, disease prevention and health promotion. So far, only few reports comprised data on domains of health literacy including Turkish migrants but without going into details (18, 20) or used very small samples (51). Health literacy among FSU migrants has mostly been neglected by research (65) and to the best of our knowledge this has not yet been reported by health literacy domains so far. Third and lastly, we explore new mechanisms by testing the explanatory power of self-efficacy and interethnic contacts for migrants' health literacy.

### Health literacy in general and in three different domains

Our results indicate a rather low general health literacy among migrants from Turkey and the former Soviet Union. Nevertheless, it is important to note that the average general health literacy score of this sample is similar to recent observations of the general population in Germany (8). Thus, migrants' health literacy in this sample is not as low as previous international research on different migrant populations indicated (10, 11, 13, 16, 17, 19). However, a particularly low health literacy is found within subgroups of the migrant population. This is an important finding that was missing so far for Germany.

In the next step, we took a deeper look at the three domains of health-related information, namely health care, disease prevention and health promotion. Health promotion literacy was lowest in our sample. This is in line with findings for the general population in various countries including Germany (4, 5, 28, 33, 49, 50, 66). However, in our study health promotion literacy is closely followed by disease prevention literacy and both are substantially lower than health care literacy. Thus, dealing with information regarding health promotion and prevention in our sample is perceived to be more difficult than health care. This is in contrast to German studies on the general population (33, 49), which showed a similar degree of health care and disease prevention literacy. An Austrian report on migrants also indicates low disease prevention and health promotion literacy (20). The low health literacy in these domains should have been alleviated by the ‘prevention act' which was passed in 2015. The law was supposed to expand on the disease prevention and health promotion measures (67). While the range of measures has grown strongly since then, user-friendly information for migrants on these topics is still lacking.

### Health literacy and migrant-specific factors

To be able to design interventions, knowledge about vulnerable groups is crucial. Therefore, associations with health literacy in the different domains were analyzed. Our results indicate differences between Turkish and FSU migrants in some domains. While we do not see any ethnic contrasts for general health literacy and health promotion health literacy there are differences in the domains of health care literacy and disease prevention literacy by *region of origin*. Migrants from Turkey and their descendants seem to have a substantially higher health care but lower disease prevention literacy than migrants from former Soviet Union. While the deviation in health care literacy between both immigration groups already disappears when taking generation into account, Turkish migrants still have a lower disease prevention literacy after controlling for differences in demographic and socioeconomic factors, literacy, chronic illness and self-efficacy. Migrants from Turkey apparently face more challenges in dealing with disease prevention information, including information tasks on vaccination, preventive check-ups, medical screenings and dealing with health risks in general than FSU migrants, who resemble the general population in Germany in this domain (33). This difference may (partly) be explained by differences the health care systems and attitudes toward prevention and disease patterns in the countries of origin. For example, the Semashko-System in the FSU already provided a good basic care in a standardized system, open for everyone, and included public health measures such as prevention, hygiene and other preventive measures. This only changed in the late 1980s and became weaker after the fall of the FSU (68). In Turkey, preventive measures were also provided, however, the focus is more on health care and therapy than on preventive measures in the system (69). Turkish migrants have been described to focus more on treating illness rather than making use of preventive measures (70). In the German health system, a lot of disease prevention measures, like common vaccinations and disease-specific screenings, are financed by the statutory health insurance and are therefore cost free for users. Since the passing of the so-called Prevention Act in 2015, disease prevention and health promotion received more attention (71). Experiences with preventive measures in the country of origin seem to shape migrants' expectations and influence their behavior in the receiving country. The amount of difficulties they face with information about prevention measures might be higher if those were not common in their country of origin. To the best of our knowledge, this has not been studied so far. Further research is needed to investigate the relevance of country of origin and differences in health care systems and attitudes for health literacy in different domains.

Previous studies also reported lower health literacy for the first *migration generation* (12, 21, 52) and those with a shorter *duration of stay* [ex. (15, 16, 32, 45, 46)]. Our two groups of study have different migration histories. The Turkish immigration group has been in Germany for many decades and is thus composed of different migration generations, while most FSU migrants belong to the first generation (53). We found strong evidence that descendants of migrants, i.e., persons that were born in Germany, have substantially higher health literacy in all domains than first generation migrants. However, generational differences can largely be explained by demographic and socioeconomic variables except for respondents with a short duration of stay (up to 5 years) who have a significantly lower general health literacy and health care literacy compared to second-generation migrants. No effect of duration of stay was found for disease prevention and health promotion when taking demographic aspects into account. This indicates that recent immigrants including refugees are more vulnerable when dealing with information about diseases, their management and the interaction with medical professionals and the health care system. This seems plausible as their knowledge of the health care system is rather limited shortly after they arrive and grows with a longer stay in the receiving country. This also means that there is a need for the provision of information on health care for migrants who recently migrated to reduce the disparities in difficulties dealing with health care related information tasks.

### Health literacy and demography and socioeconomy

Besides country of origin and duration of stay, socioeconomic variables contributed toward explaining health literacy. In sum, *low educational level, low social status and financial deprivation* were negatively correlated with health literacy. This is in line with German and international health literacy research in general [ex. (4, 6, 8, 9, 22, 28, 29)] and in migrant populations (12, 32, 45). It is also noteworthy that adding demographic and socioeconomic determinants to the model considerably decreases differences by region of origin. In Germany, migrants tend to have a lower socioeconomic position [ex. (72)] and thus part of the variation in health literacy is due to socioeconomic disadvantages. Interventions need to focus on socioeconomically disadvantaged groups and create measures to make information more accessible, understandable and applicable for persons with poor education, financial deprivation and low social status. However, the socioeconomic disadvantage is not the only explanation for low health literacy.

In addition, we have shown that *age* is a relevant predictor of health care literacy and health promotion literacy. In contrast, we could not observe an association in the domain of disease prevention. This resembles findings for the general population sample in Germany (33). Results from other countries investigating the association of age and general health literacy have been inconsistent [ex. (25)]. We conclude, that it is necessary to differentiate by domains and that this focus might lead to more consistent findings. Our results imply that there is a need to focus on older people in health care information interventions but not in disease prevention information interventions in Germany. As in the general population in Germany (33), *gender* differences were found for general health literacy, disease prevention literacy and health promotion literacy, and were higher among female migrants. This is plausible as migrant women sometimes manage health matters for family members (64). No substantial deviation from male migrants was found for health care literacy when controlling for other characteristics. However, it has to be noted that gender does only account for small variations of health literacy in our study. Age and gender should be considered when designing interventions. Strategies to design and promote health information should be adjusted to different age-groups and among men and women, especially in different domains. The bivariate results also show that migrants with at least one *chronic disease* face more challenges with health information in the domains of health care and health promotion, but not in the regression analyses. In contrast, having a chronic disease was associated with a lower health literacy among the general population in Germany across all domains (33). This needs further exploration in future studies.

Low health-related literacy skills were also strongly associated with lower health literacy across domains. The effect of low proficiency in the receiving country's language on health literacy is not surprising and has been well documented for migrants [ex. (10, 12, 15, 16, 32, 44, 46). Thus, there is a need for information in a simple language. It also needs to be reflected if information should be provided in more accessible formats such as videos or pictures.

Our study adds to the scare literature of determinants on health literacy in domains by showing that the associations are mostly constant across the three domains of health literacy.

### Health literacy and self-efficacy

Our results add to the state-of-knowledge on health literacy by considering self-efficacy using a migrant sample. The results show that migrants with higher self-efficacy had better health literacy than migrants with lower self-efficacy in regression models across all domains. Hence, migrants with strong beliefs in their own capabilities “to organize and execute the courses of action required to produce given attainments” (34) perceived tasks in dealing with health relevant information in different domains as less difficult. This resembles findings in the general population and children in Germany (35, 73). Thus, strengthening self-efficacy might positively affect health literacy. This might also prevent reverse effects if difficulties in dealing with health information might negatively affect self-efficacy.

### Health literacy and interethnic contact

Previous findings emphasized the importance of the social context for health literacy, especially among vulnerable groups (39, 41–43, 64). We included interethnic contacts as a form of social ties and social integration. Previous research has repeatedly emphasized the importance of interethnic ties as generators of “bridging social capital” for ethnic minority integration that can equip migrants with resources relevant in the country of residence [ex. (74, 75)]. For the first time, we were able to show quantitatively that interethnic contacts might help migrants dealing with health-related information. In our study, we find that interethnic contacts with native Germans are positively linked to a higher health literacy across all domains except for disease prevention. Social ties with persons that are familiar with the national health system seem to be important to gain relevant information on health–related aspects in the receiving country. These ties could also be a resource or even compensation for the above-mentioned possible disadvantages, like lower socioeconomic position or literacy skills. We suspect that these ties play less of a role for disease prevention, as the occurrence of diseases is rather unforeseeable and therefore triggers insecurity among all parts of the population. In the COVID-19 pandemic, it became clear, that prevention knowledge changes at a much faster pace compared, for instance, to knowledge about the health care system, which is a more static entity. This in turn will affect the degree to which it can be transmitted through personal ties; knowledge that changes at a faster pace cannot be transmitted as efficiently. We argue that interethnic contact is therefore more important for all other domains of health literacy. However, information must also be designed and provided in a better way to meet the needs of socially less integrated migrants and, for instance, additionally train persons from the community as health information multipliers/mediators (76). Further research should examine the social support within interethnic contacts.

### Limitations

Limitations arise primarily in two areas: the area of sampling and the area of measurement of health literacy. First, due to the COVID-19 pandemic and difficulties with face-to-face surveys in respondents' households, we relied on community-based sampling. This is strictly speaking not a probability sample and therefore hampers the representativity of our sample. This is reflected in a higher share of highly educated migrants in both immigration groups, higher share of older persons in the FSU sample and higher share of young persons in the Turkey sample compared to official statistics (53, 77). Due to quotas for each immigration group, the sample consists of similar shares of persons who migrated to Germany and descendants of migrants as well as similar shares of males and females compared to the target population. German citizenship was overrepresented in the Turkey sample and underrepresented in the FSU sample, while the share of persons with a shorter duration of stay in Germany was higher than in the target population in both samples. Hence, we need to be cautious with the interpretation of descriptive statistics. However, in regression analyses we control for education and other variables, partially accounting for this sampling bias. Other studies could also show that it can be beneficial to use non-probability sampling methods especially in the immigrant population since more vulnerable subgroups can be included in the survey (78–81). The second limitation occurs with regard to the measurement of our dependent variable health literacy. The scores are based on self-rated difficulties with the different domains of health literacy. However, perceptions can often be biased. For instance, some respondents might underestimate difficulties, whereas others overestimate their difficulties. Especially for migrants, research on educational aspirations has repeatedly shown that some immigration groups are more optimistic about their skills than natives [ex. (82–84)]. To buffer this bias, our models held actual health-related literacy skills constant. Furthermore, the calculated health literacy scores following international procedure (6) suffer from loss of information due to the dichotomization of the four-point item answer options. However, the scores still give a useful impression of the individual's health literacy in the light of the naturally superficial limitations of quantitative studies. Health-related literacy skills in this study were measured using the NVS test and the food label was provided in German. Although this test measures a small set of literacy skills related to health, i.e., nutrition, we believe that this more objective measure reflects literacy skills in German language better than the first language of the respondents or self-assessed measures on German language proficiency. Last, interethnic contact might interact with self-efficacy. Hence, coefficients may be biased. This would need further exploration in future studies. All in all, we believe that our study has contributed to the existing state-of-the-art on health literacy by (a) conducting the first study on Turkish and FSU migrants in Germany, (b) investigating domain-specific health literacy and (c) considering novel factors associated with health literacy such as self-efficacy and interethnic contact.

## Conclusion

Our study draws attention to the importance of migrant's health literacy. We show that health information on health promotion and disease prevention are more difficult to process than information regarding health care. Therefore, measures addressing the provision of information (e. g. enhancing accessibility and usability of health information) need to take differences between the domains into account. We also reveal that migrants in this study cannot generally be considered as vulnerable, as often outlined, but there are subgroups of the migrant population that have lower health literacy. There is need for targeted interventions, especially for socioeconomically disadvantaged, older migrants, those with poor German language skills, and recently migrated. In addition, self-efficacy and poor interethnic contact need to be addressed to reduce inequalities in health literacy.

## Data availability statement

The raw data supporting the conclusions of this article will be made available by the authors upon request, without undue reservation.

## Ethics statement

The studies involving human participants were reviewed and approved by Ethics Committee of Bielefeld University (EUB-2019-104). Written informed consent for participation was not required for this study in accordance with the national legislation and the institutional requirements.

## Author contributions

DS and E-MB conceived the idea of the study. The analyses were performed by JK and E-MB. The draft of the manuscript was written by E-MB, JK, and SC. DS commented and edited versions of the manuscript. All authors contributed to the study design and questionnaire development, interpreted the data and have read and approved the final manuscript.

## Funding

This work was funded by Robert Bosch Foundation (grant number 01000081-001). The funders have had no influence on the study design, data collection and analysis, interpretation of the results, the manuscript and the decision to submit it for publication.

## Conflict of interest

The authors declare that the research was conducted in the absence of any commercial or financial relationships that could be construed as a potential conflict of interest.

## Publisher's note

All claims expressed in this article are solely those of the authors and do not necessarily represent those of their affiliated organizations, or those of the publisher, the editors and the reviewers. Any product that may be evaluated in this article, or claim that may be made by its manufacturer, is not guaranteed or endorsed by the publisher.
