# Supplementary material for: Differences in health literacy domains among migrants and their descendants in Germany
Source: Front Public Health. 2022 Sep 23;10:988782. doi: 10.3389/fpubh.2022.988782 (PMC9541527; doi:10.3389/fpubh.2022.988782)
Supplement: Supplementary file 1 [file Table_1.pdf]

# *Supplementary Material*

**Supplementary Table 1.** Comparison of sample characteristics of both immigration groups in the sample

| | Turkish migration background | FSU migration background |
|---|---|---|
| **n** | 412 | 413 |
| **Duration of stay** | | |
| *mean, min–max* | *30.7, 0-58* | *17.5, 0-46* |
| 5 years and below | 5.1% | 18.9% |
| 6–25 years | 17.0% | 51.3% |
| 26 years and longer | 32.5% | 20.6% |
| Born in Germany | 45.4% | 9.2% |
| **Age** | | |
| *mean, min–max* | *40.3, 18-88* | *46.7, 18-91* |
| 18–29 years | 34.5% | 14.5% |
| 30–45 years | 27.4% | 40.0% |
| 46–64 years | 29.9% | 28.8% |
| 65 years and older | 8.3% | 16.7% |
| **Educational level** | | |
| Low (ISCED 0–2) | 31.3% | 13.6% |
| Medium (3–4) | 44.4% | 36.3% |
| High (5–8) | 24.3% | 50.1% |
| **Financial deprivation** | | |
| None | 35.0% | 12.3% |
| Low | 51.0% | 56.2% |
| Medium–high | 14.1% | 31.5% |
